# Dynamic Changes of Post-Translationally Modified Forms of CXCL10 and Soluble DPP4 in HCV Subjects Receiving Interferon-Free Therapy

**DOI:** 10.1371/journal.pone.0133236

**Published:** 2015-07-16

**Authors:** Eric G. Meissner, Jérémie Decalf, Armanda Casrouge, Henry Masur, Shyam Kottilil, Matthew L. Albert, Darragh Duffy

**Affiliations:** 1 Division of Infectious Diseases, Department of Microbiology and Immunology, Medical University of South Carolina, Charleston, SC, United States of America; 2 Laboratory of Immunoregulation, National Institute of Allergy and Infectious Diseases, NIH, Bethesda, MD, United States of America; 3 Critical Care Medicine Department, NIH, Bethesda, MD, United States of America; 4 The Laboratory of Dendritic Cell Immunobiology, Institut Pasteur, Paris, France; 5 INSERM U818, Paris, France; 6 Institute of Human Virology, University of Maryland School of Medicine, Baltimore, MD, United States of America; 7 Center for Human Immunology, Institut Pasteur, Paris, France; University of Washington, UNITED STATES

## Abstract

Serum levels of the interferon (IFN)-stimulated chemokine CXCL10 are increased during chronic HCV infection and associate with outcome of IFN-based therapy. Elevated levels of NH2-terminal truncated CXCL10 (3-77aa), produced by DPP4 cleavage, negatively associate with spontaneous clearance of acute HCV infection and sustained virological response (SVR) with IFN-based therapy for chronic infection. The association of different CXCL10 forms and DPP4 with outcome during IFN-free HCV therapy has not been examined. Using novel Simoa assays, plasma was analyzed from HCV genotype-1 (GT1) subjects who relapsed (n = 11) or achieved SVR (n = 10) after sofosbuvir and ribavirin (SOF/RBV) treatment, and from SOF/RBV relapsers who achieved SVR with a subsequent SOF/ledipasvir regimen (n = 9). While the NH2-truncated form of CXCL10 was elevated in HCV infection relative to healthy controls, pre-treatment plasma concentrations of CXCL10 forms failed to stratify subjects based on treatment outcome to IFN-free regimens. However, a trend (statistically non-significant) towards elevated higher levels of total and long CXCL10 was observed pre-treatment in subjects who relapsed. All forms of CXCL10 decreased rapidly following treatment initiation and were again elevated in subjects who experienced HCV relapse, indicating that CXCL10 production may be associated with active viral replication. While soluble DPP4 (sDPP4) and NH_2_-truncated CXCL10 concentrations were highly correlated, on-treatment sDPP4 levels and activity declined more slowly than CXCL10, suggesting differential regulation.

**Conclusion:**

These data suggest post-translationally modified forms of CXCL10 will not support the prediction of treatment outcome in HCV GT1 subjects treated with SOF/RBV.

## Introduction

Chronic HCV infection can be effectively treated with IFN-free regimens composed of direct acting antivirals (DAA) that demonstrate improved tolerability and efficacy compared to IFN-based treatment [1, 2]. HCV relapse is the most common reason for treatment failure with current IFN-free DAA regimens, although mechanisms and predictors of relapse are not well understood. Higher activation of innate and adaptive immune function during treatment has been observed in subjects achieving SVR with IFN-free DAA therapy relative to relapsers [3, 4], suggesting host immunity may impact and inform treatment outcome. Identifying biomarkers predictive of outcome could help individualize treatment durations required to achieve SVR and enable response-guided therapy where costly DAA treatments are not universally available [5, 6].

*CXCL10* is an IFN-stimulated gene (ISG) whose protein product CXCL10 is produced by hepatocytes and non-parenchymal liver cells during chronic HCV infection due to chronic inflammation [7, 8]. CXCL10 signaling through the chemokine receptor CXCR3 plays an important role in hepatic lymphocyte migration and function during HCV infection [9–12]. Levels of total CXCL10 in serum negatively associate with spontaneous clearance of acute infection and treatment outcome with IFN-based therapy [13–17].

Interestingly, enzymatic cleavage of full length CXCL10 (referred to herein as long CXCL10) by dipeptidyl peptidase-4 (DPP4, also known as CD26) generates a short NH_2_-terminal truncated form of CXCL10 (referred to herein as short CXCL10). This post-translationally modified form was not measured in most studies associating CXCL10 with HCV treatment outcome. Unique antibodies have been developed to detect long CXCL10 (amino acids 1–77) and short CXCL10 (amino acids 3–77) allowing quantitation of functional chemokine forms in clinical samples, in parallel to undefined CXCL10 forms with commercially available antibodies (total CXCL10) [18]. Studies that discriminated differential CXCL10 forms revealed that pre-treatment levels of short and total CXCL10, but not long CXCL10, associated with non-response to IFN-based therapy and clearance of acute infection [19, 20]. In addition, plasma sDPP4 levels, shown to be the predominant mediator of CXCL10 cleavage into the short form [19], were associated with non-response to IFN-based therapy and advanced liver disease [21, 22]. Taken together, these data suggest that generation of short CXCL10 is a biologically significant post-translational modification that impacts and informs outcome with IFN-based therapy.

Exact quantification of the different CXCL10 forms from clinical samples has been restricted due to the limit of detection achieved with standard ELISA and Luminex assays. Recently developed Single Molecule Array (Simoa) technology (Quanterix) overcomes these limitations and provides increased sensitivity for protein biomarker quantification compared to conventional immunoassays [23]. In the present study, we tested clinical samples using Simoa assays to evaluate how CXCL10-associated biomarkers changed during IFN-free DAA therapy and analyzed associations with treatment outcome.

## Materials and Methods

### Subjects and samples

Plasma was collected from subjects treated with SOF/RBV on the SPARE trial (NCT01441180), conducted at the National Institute of Allergy and Infectious Diseases (NIAID), and stored at -80°C until testing. In this trial, treatment naïve subjects chronically infected with HCV GT1 received 24 weeks of SOF (400 mg daily) combined with low-dose (600 mg daily) or weight-based (1000–1200 mg daily) RBV [24]. Fourteen of seventeen subjects who relapsed after SOF/RBV treatment in the SPARE trial were subsequently treated with SOF/ledipasvir (400/90 mg daily, fixed-dose combination) for 12 weeks in the NIH SYNERGY trial (NCT01805882). The NIH/NIAID Institutional Review Board (IRB) approved both studies, all subjects provided written informed consent documented on IRB-approved consent forms, and the studies were conducted in accordance with the Declaration of Helsinki. Based on sample availability, pre-treatment and on-treatment (day 6–11 and week 20) cryopreserved plasma was analyzed from 10 subjects who achieved SVR and 11 subjects who relapsed in the SPARE trial. Cryopreserved plasma from 9 SPARE relapsers who were subsequently treated in the SYNERGY trial was analyzed at pre-treatment (post-relapse with SOF/RBV) and on-treatment (week 8) time points. Plasma from healthy volunteers was provided by the Etablissement Français du Sang (EFS) in Paris, and was screened as being HCV/HBV/HIV negative. Blood was collected on heparin and plasma was obtained after centrifugation.

### CXCL10 quantification

Plasma concentration of total (R&D clone 33036), long (1-77aa), and short (3-77aa) CXCL10 forms was measured using Simoa technology (Quanterix). Homebrew Simoa assays specific for the three CXCL10 forms were prepared following the manufacturer’s recommendations using antibody pairs previously described [18]. The limits of detection (LOD) were 1 pg/ml for long and short CXCL10 assays and 0.05 pg/ml for total CXCL10 (S1A Fig). CXCL10 forms were also quantified using standard sandwich ELISA, as previously described [19].

### sDPP4 quantity and activity

Levels of sDPP4 were measured using the human DPP4 ELISA (R&D) and enzymatic activity was determined with the luciferase-based DPP4-Glo protease assay (Promega, Southampton, UK) according to the manufacturer’s instructions, with recombinant DPP4 used as a reference (Sigma ref D4943).

### Statistical analysis

All plasma samples were run in triplicate to determine a mean value. Statistical analysis and graphical presentation was performed using GraphPad Prism 6.0 software. Data analysis used non-parametric assumptions. Spearman correlations greater than 0.4 were considered significant.

## Results

To explore the dynamic expression of CXCL10 forms and sDPP4 activity during IFN-free DAA therapy, we tested longitudinal plasma samples collected from subjects treated with SOF/RBV for 24 weeks and from SOF/RBV relapsers who subsequently achieved SVR with SOF/ledipasvir. The patient cohorts had a high prevalence of African-American subjects and unfavorable IL28B genotype (non-CC), risk factors for non-response with IFN-based therapy [24, 25]. In the SPARE trial, 17 of 55 subjects who completed treatment experienced relapse [24]. Of these 17 subjects who relapsed, 14 were enrolled in the SYNERGY study and treated with SOF/ledipasvir, all of whom achieved SVR [25].

We previously observed a rapid decline in plasma CXCL10 which correlated with viral kinetic decline in the SPARE trial, with levels of total CXCL10 declining to end of treatment levels by 1 week of treatment [3]. There was a trend (p = 0.08) towards higher pre-treatment CXCL10 levels in subjects who later experienced treatment relapse, while no other differences in on-treatment CXCL10 decline or levels based on treatment outcome were detected [3]. DPP4 gene expression did not change in liver or blood over the course of treatment based on paired microarray analysis, while CXCL10 gene expression was strongly down-regulated in both blood and liver with therapy ([3] and data not shown).

**Fig 1:**
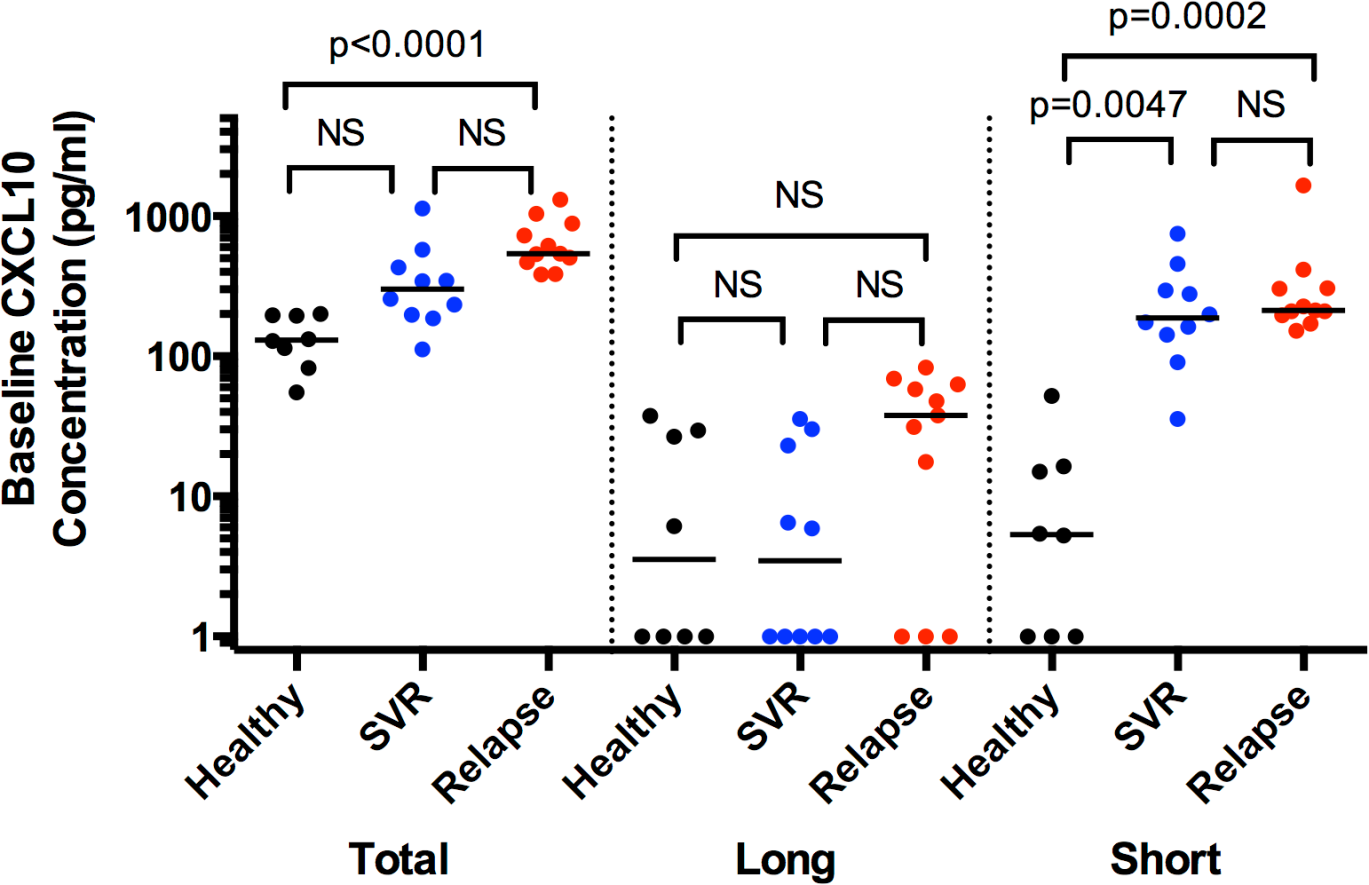
CXCL10 forms do not differ pre-treatment in patients who achieve SVR vs. relapse after SOF/RBV treatment. Plasma collected pre-treatment from 21 subjects (SVR = 10, relapse = 11) treated with SOF/RBV in the SPARE trial and 8 healthy controls was analyzed for total, long, and short forms of CXCL10 using Simoa. Analysis is by Kruskal-Wallis with a multiple test correction. Shown are individual values and medians. NS = not significant (p>0.05), SVR = sustained virologic response, Rel = relapser.

**Fig 2:**
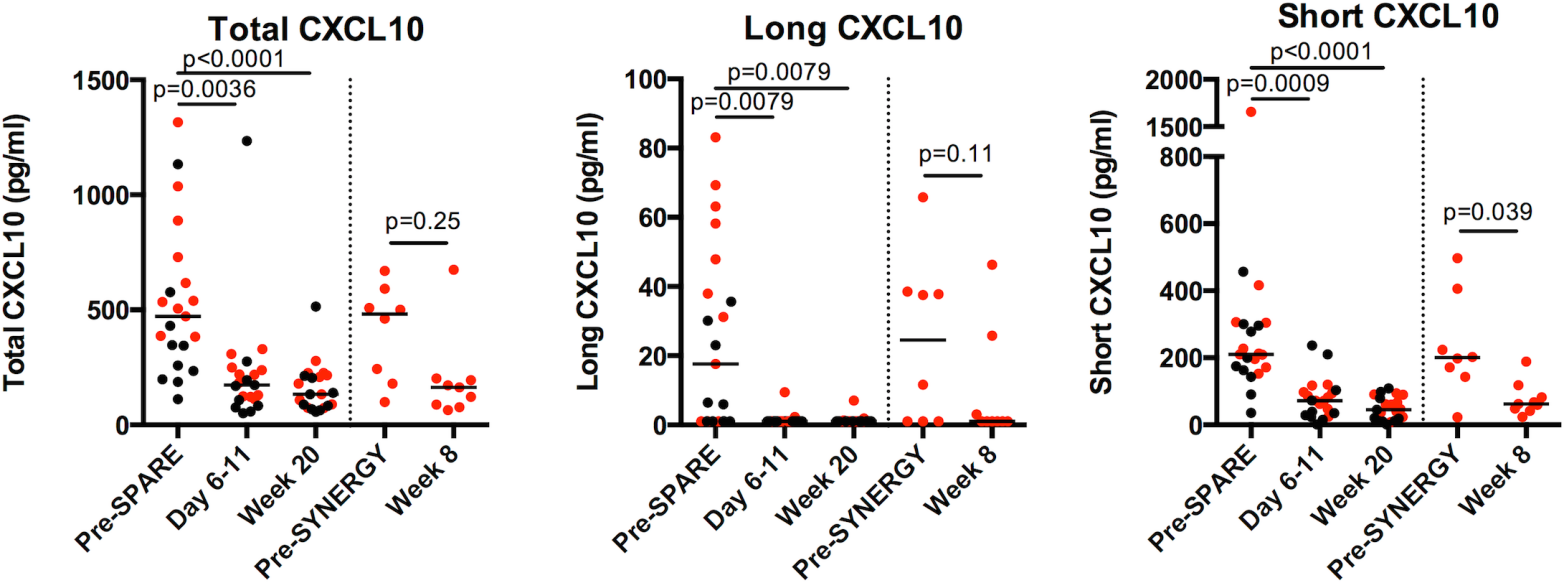
CXCL10 forms decline rapidly during IFN-free therapy with SOF/RBV. Plasma collected pre-treatment, day 6–11 of treatment, and at week 20 from 21 subjects (SVR = 10, relapse = 11) treated with SOF/RBV in the SPARE trial was analyzed for total, long, and short forms of CXCL10. For SPARE relapsers, 9 subjects who were treated with SOF/ledipasvir (SYNERGY trial) were assayed pre-treatment and at week 8. Longitudinal changes in total (A), long (B), and short (C) forms are displayed for subjects achieving SVR (black circles) and for relapsers (red circles). Statistical analysis is by Kruskal-Wallis with a multiple test correction considering data from all subjects (SPARE data before dotted line) or Wilcoxon paired test (SYNERGY data after dotted line). Assays were run in technical triplicates. Shown are individual values and medians.

In the present study, we analyzed samples available from 21 subjects treated with SOF/RBV for in depth analysis of different CXCL10 forms, hypothesizing that short CXCL10 may serve as a potential biomarker of treatment outcome. We utilized the Simoa platform, which allowed for more sensitive detection of CXCL10 forms from patient samples than standard ELISA (lower limit of detection 0.5–1.0 pg/ml, S1 Fig). We found no significant difference in pre-treatment levels of total, long, and short CXCL10 between subjects achieving SVR compared to relapsers, with both groups manifesting higher short CXCL10 levels relative to healthy controls (Fig 1). A trend towards elevated total (p = 0.09) and long (p = 0.09) CXCL10 concentration pre-treatment in relapsers relative to subjects achieving SVR was observed, while no such trend was observed for short CXCL10 (p>0.99). Similar to total CXCL10, both long and short forms declined rapidly on SOF/RBV therapy to levels observed in healthy controls, were re-induced upon virologic relapse, and declined again upon treatment with SOF/ledipasvir (Fig 2A–2C). No differences in on-treatment decline of any CXCL10 forms were detected between subjects who relapsed vs. achieved SVR. Furthermore, no difference in viral load was observed at day 0 or day 7 in this subset of subjects based on treatment outcome, with 19 of 21 patients having less than 2 logs of detectable HCV in serum by week 1. These data indicate an association between normalization of plasma CXCL10 concentration with treatment duration, which corresponds to rapid treatment-induced viral clearance.

**Fig 3:**
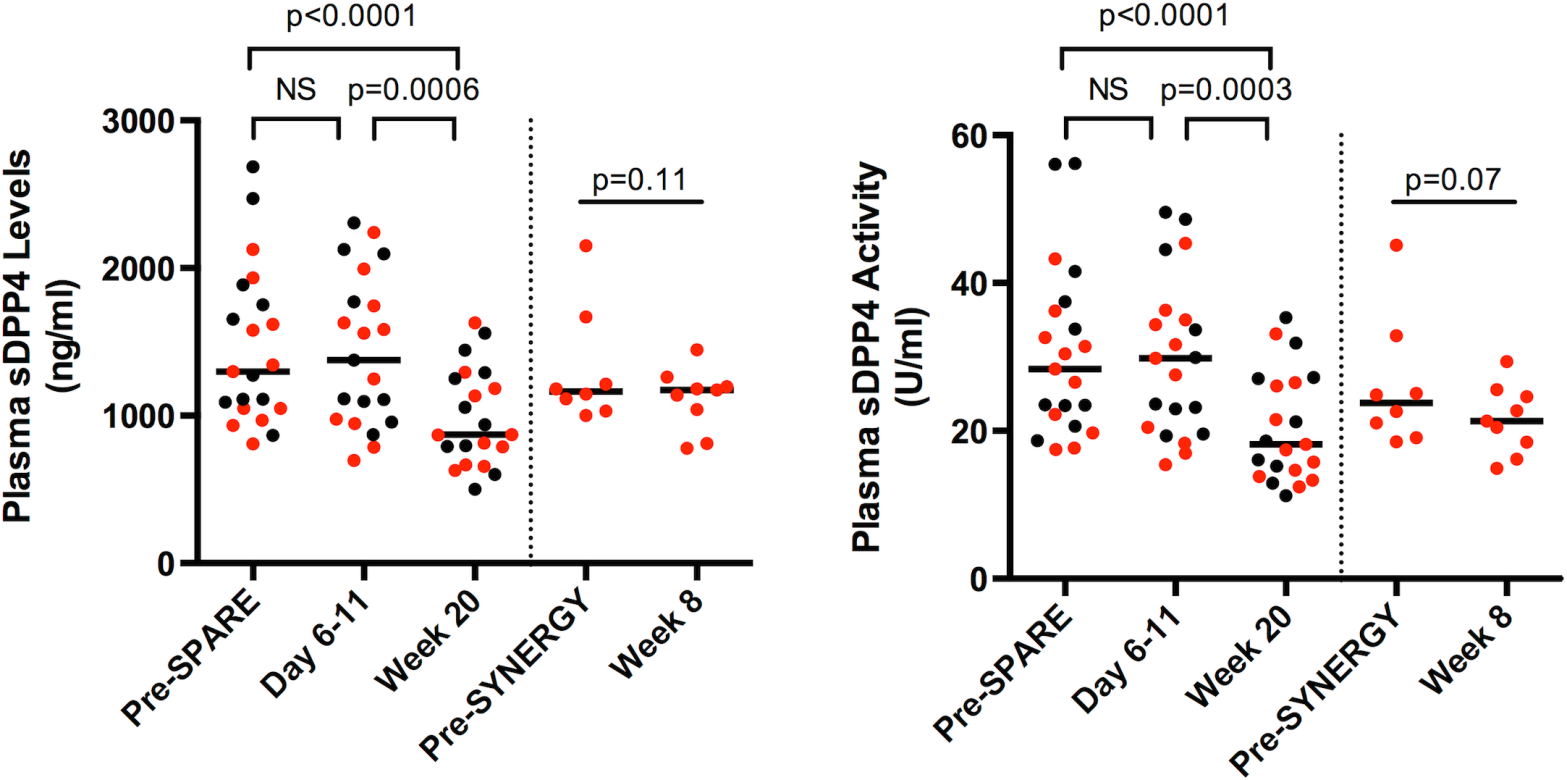
sDPP4 activity and levels decline during treatment with slower kinetics than CXCL10 forms. sDPP4 levels (A) and activity (B) were determined longitudinally on samples from subjects as described in Fig 2. Data from subjects achieving SVR (black circles) and those who relapsed (red circles) on SPARE are shown. Statistical analysis is by Kruskal-Wallis with a multiple test correction considering data from all subjects (SPARE data before dotted line) or Wilcoxon paired test (SYNERGY data after dotted line). Assays were run in technical triplicates. Shown are individual values and medians.

DPP4 is believed to be the primary enzyme mediating truncation of long CXCL10 to produce short CXCL10 *in vivo* [19]. We asked whether pre- or on-treatment sDPP4 activity and levels could serve as a biomarker of outcome, but detected no difference in pre-treatment or on-treatment sDPP4 levels or decline (Fig 3A and 3B). Interestingly, sDPP4 levels and activity remained elevated at week 1 of treatment, despite all CXCL10 forms having returned to healthy levels, suggesting differential disease-associated modulation of sDPP4 and plasma CXCL10. By week 20 of treatment, a decline in sDPP4 activity and levels was apparent (Fig 3). In the 9 patients we analyzed who were treated with SOF/ledipasvir after SOF/RBV relapse, pre-SPARE and pre-SYNERGY treatment values for sDPP4 activity and levels did not differ significantly (Fig 3, analysis not shown).

**Fig 4:**
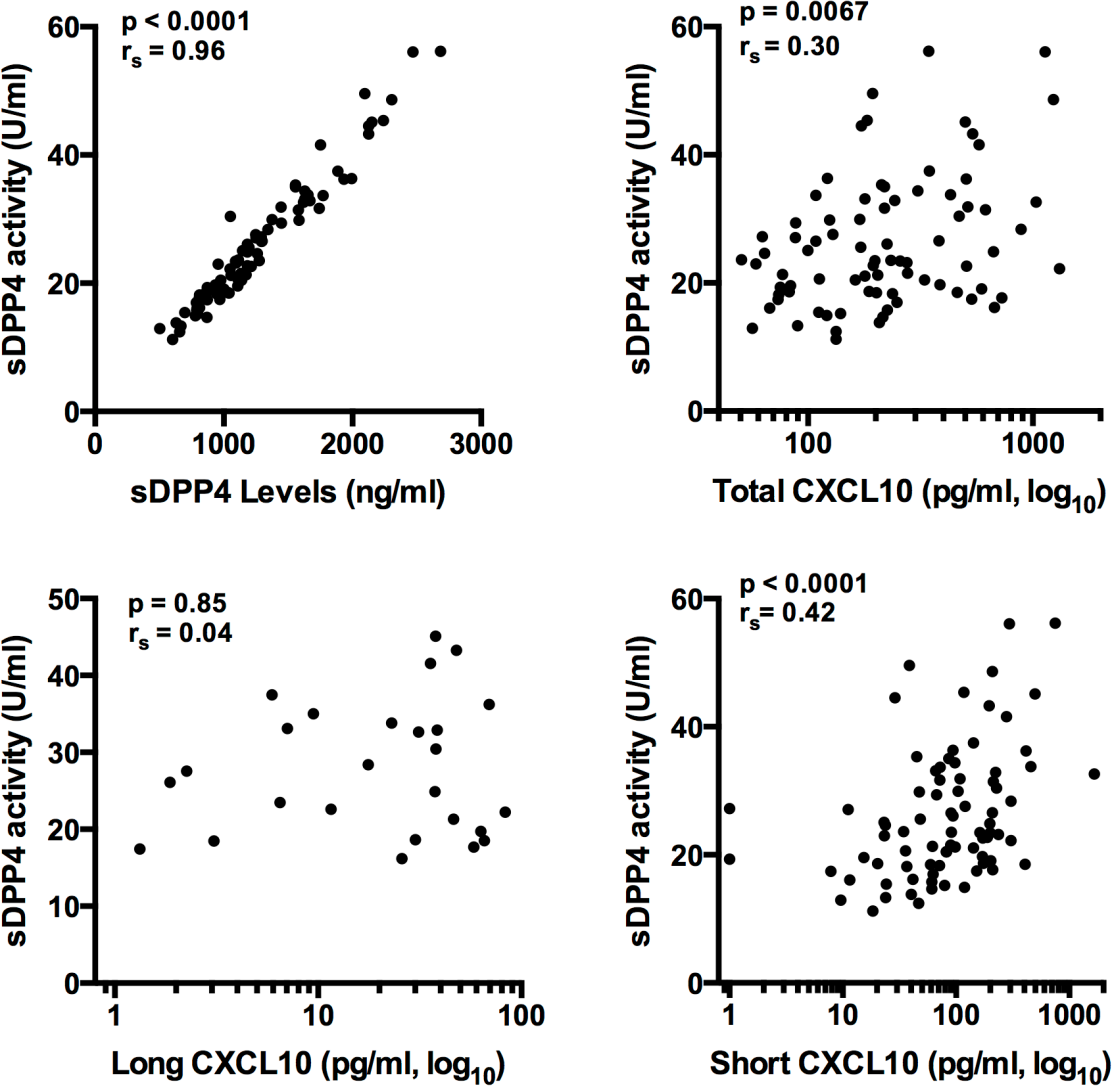
Total and short CXCL10, but not long CXCL10, correlate with sDPP4 activity. (A) Correlation between DPP4 levels and activity for all subjects and all time points tested (n = 80). (B-D) Correlation of total, long, and short CXCL10 forms with DPP4 activity. For long CXCL10, only data from samples with detectable long CXCL10 (above LOD of 1 pg/ml) were used for analysis (n = 26). Spearman correlation (r_s_) was determined for each analysis.

To further address the hypothesis that DPP4 is the primary mediator of short CXCL10 production *in vivo*, we correlated expression levels of sDPP4 and CXCL10 forms. sDPP4 levels and activity were strongly correlated (Fig 4), with no difference in this correlation based on treatment response (data not shown). A correlation was observed between short CXCL10 and sDPP4 levels (r_s_ = 0.42, p<0.0001) (Fig 4). A weaker correlation was observed with total CXCL10 (r_s_ = 0.30, p = 0.0067), and no correlation was observed with long CXCL10. Additionally, we did not detect an association of pre-treatment CXCL10 or sDPP4 levels with hepatic inflammation (plasma ALT) or baseline HCV viral load (r_s_ < 0.4, data not shown).

## Discussion

This study has taken advantage of novel ultra-sensitive immunoassays to examine the dynamics of CXCL10 forms and sDPP4 expression during IFN-free therapy for chronic HCV infection. While we demonstrate a direct correlation between plasma sDPP4 and circulating forms of NH_2_-truncated CXCL10 in chronic HCV infection, no associations were found for SOF/RBV treatment outcome based on analysis of a cohort of 11 subjects who relapsed and 10 subjects who achieved SVR. These data, in failing to reject the null hypothesis, suggest that CXCL10 and sDPP4 are not viable biomarkers for predicting treatment outcome for IFN-free regimens.

Nonetheless, the data obtained provides unique insight into the host immune response during SOF/RBV and SOF/ledipasvir therapy. Specifically, we show that immune perturbations induced by chronic viral infection can be rapidly reset following therapeutic intervention with DAAs, and provide insight into the regulation of sDPP4 activity and CXCL10 forms. Higher levels of short CXCL10 were observed in HCV-infected subjects relative to healthy controls, indicating an infection-associated post-translational modification of this chemokine. As sDPP4 levels also declined with HCV treatment, this suggests that sDPP4 and short CXCL10 are both elevated during chronic infection and decrease upon inhibition of viral replication with DAAs.

Up-regulation or shedding of DPP4 and enzymatic generation of short CXCL10 are consistent with the concept that chemokine dysfunction may contribute to an ineffective immune response in chronic viral infection [19]. One interesting observation was the different on-treatment kinetic decline of sDPP4 activity and CXCL10 concentration. While both are strongly associated with chronic hepatitis, this observation suggests differential mechanisms of regulation following viral clearance. CXCL10 is likely induced by type-I and type-III IFNs triggered by HCV replication. By inhibiting viral replication, *CXCL10* gene expression, and other interferon-stimulated genes, decline rapidly. While DPP4 has not been reported to be an interferon-stimulated gene, its level of expression has been previously associated with liver fibrosis [21]. Together, these observations suggest that while CXCL10 may directly reflect HCV replication, sDPP4 regulation and/or shedding may be indirectly affected through disease-induced liver inflammation. Such inflammation-induced metabolic dysregulation may be responsible for elevated sDPP4 and as such, treatment-induced decline may signal a second step towards restoring virus-free homeostasis. An alternative explanation may be that differences in plasma stability of CXCL10 and sDPP4 impact the differential rate of normalization. New insight into the post-translational modifications of sDPP4 may permit discrimination of newly synthesized from existing pools of protein.

With increasing efficacy of IFN-free therapies, the need for predictive biomarkers has diminished, although there may be value in surrogate biomarkers that can guide treatment duration as a means to reduce cost while maintaining high rates of SVR. One potential limitation of this study was the relatively small sample size of patients examined. In this regard, the non-significant trend towards higher total and long CXCL10 observed in relapsers pre-treatment may become more apparent with a larger sample size. The potential association of CXCL10 forms or sDPP4 with treatment outcome could be addressed in larger studies as IFN-free treatments become more widely available.

## Supporting Information

S1 Fig Standard curves obtained for the total, long and short CXCL10 Simoa assays. (A) Concentrations of recombinant CXCL10 forms were measured using the Simoa assay. Each curve was generated from 5 independent experiments run in triplicate and compared with standard curves obtained with previously described ELISA assays [19]. For Simoa assays (filled circles), mean Average Enzyme per Bead (AEB) are shown on the left axis. For ELISA assays (open circles), absorbance at 450 nm (Abs) is shown on the right Y-axis. Limits of detection (LOD) of each assay are indicated on the graph, showing improved sensitivity of Simoa assays. (B) Correlation between plasma CXCL10 using standard ELISA vs. the Simoa platform (n = 80). Eleven samples with unquantifiable CXCL10 on the standard ELISA that had quantifiable levels detected using Simoa are shown in red. Spearman correlation (r_s_) between the assay results is shown.
(TIF)
