# Correction: Dynamic Changes of Post-Translationally Modified Forms of CXCL10 and Soluble DPP4 in HCV Subjects Receiving Interferon-Free Therapy

**DOI:** 10.1371/journal.pone.0139818

**Published:** 2015-09-29

**Authors:** The PLOS ONE Staff

Due to underlying tagging issues in the article XML, the abstract is being corrected to remove the "Conclusion" subheading. The publisher apologizes for the error. Please see the correct Abstract here:

## Abstract

Serum levels of the interferon (IFN)-stimulated chemokine CXCL10 are increased during chronic HCV infection and associate with outcome of IFN-based therapy. Elevated levels of NH2-terminal truncated CXCL10 (3-77aa), produced by DPP4 cleavage, negatively associate with spontaneous clearance of acute HCV infection and sustained virological response (SVR) with IFN-based therapy for chronic infection. The association of different CXCL10 forms and DPP4 with outcome during IFN-free HCV therapy has not been examined. Using novel Simoa assays, plasma was analyzed from HCV genotype-1 (GT1) subjects who relapsed (n = 11) or achieved SVR (n = 10) after sofosbuvir and ribavirin (SOF/RBV) treatment, and from SOF/RBV relapsers who achieved SVR with a subsequent SOF/ledipasvir regimen (n = 9). While the NH2-truncated form of CXCL10 was elevated in HCV infection relative to healthy controls, pre-treatment plasma concentrations of CXCL10 forms failed to stratify subjects based on treatment outcome to IFN-free regimens. However, a trend (statistically non-significant) towards elevated higher levels of total and long CXCL10 was observed pre-treatment in subjects who relapsed. All forms of CXCL10 decreased rapidly following treatment initiation and were again elevated in subjects who experienced HCV relapse, indicating that CXCL10 production may be associated with active viral replication. While soluble DPP4 (sDPP4) and NH_2_-truncated CXCL10 concentrations were highly correlated, on-treatment sDPP4 levels and activity declined more slowly than CXCL10, suggesting differential regulation. **Conclusion:** These data suggest post-translationally modified forms of CXCL10 will not support the prediction of treatment outcome in HCV GT1 subjects treated with SOF/RBV.
