# Supplementary figures and images for: Dynamic Changes of Post-Translationally Modified Forms of CXCL10 and Soluble DPP4 in HCV Subjects Receiving Interferon-Free Therapy
Source: PLoS One. 2015 Jul 16;10(7):e0133236. doi: 10.1371/journal.pone.0133236 (PMC4504464; doi:10.1371/journal.pone.0133236)

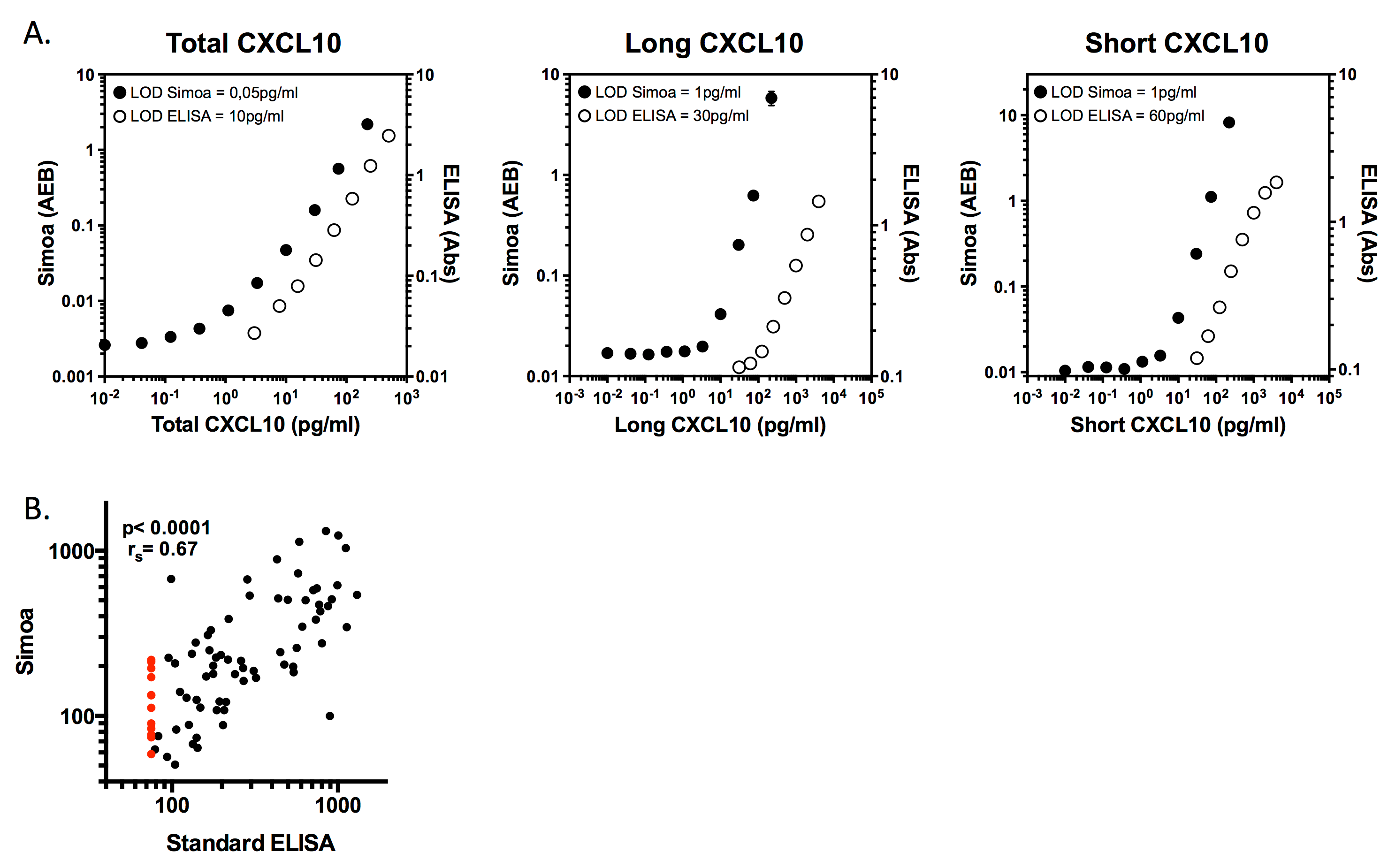

Supplement: S1 Fig — (A) Concentrations of recombinant CXCL10 forms were measured using the Simoa assay. Each curve was generated from 5 independent experiments run in triplicate and compared with standard curves obtained with previously described ELISA assays [19]. For Simoa assays (filled circles), mean Average Enzyme per Bead (AEB) are shown on the left axis. For ELISA assays (open circles), absorbance at 450 nm (Abs) is shown on the right Y-axis. Limits of detection (LOD) of each assay are indicated on the graph, showing improved sensitivity of Simoa assays. (B) Correlation between plasma CXCL10 using standard ELISA vs. the Simoa platform (n = 80). Eleven samples with unquantifiable CXCL10 on the standard ELISA that had quantifiable levels detected using Simoa are shown in red. Spearman correlation (rs) between the assay results is shown. (TIF) [file pone.0133236.s001.tif]
